# Deconvoluting Protein (Un)folding Structural Ensembles Using X-Ray Scattering, Nuclear Magnetic Resonance Spectroscopy and Molecular Dynamics Simulation

**DOI:** 10.1371/journal.pone.0125662

**Published:** 2015-05-06

**Authors:** Alexandr Nasedkin, Moreno Marcellini, Tomasz L. Religa, Stefan M. Freund, Andreas Menzel, Alan R. Fersht, Per Jemth, David van der Spoel, Jan Davidsson

**Affiliations:** 1 Department of Chemistry-Ångström laboratory, Uppsala University, Box 523, SE-75110 Uppsala, Sweden; 2 Uppsala Center for Computational Chemistry, Science for Life Laboratory, Department of Cell and Molecular Biology, Uppsala University, Box 596, SE-75124 Uppsala, Sweden; 3 Department of Physiology and Biophysics, Case Western Reserve University, Cleveland, Ohio 44106, United States; 4 Medical Research Council Laboratory of Molecular Biology, Cambridge CB2 0QH, United Kingdom; 5 Paul Scherrer Institut, 5232 Villigen-PSI, Switzerland; 6 Department of Medical Biochemistry and Microbiology, Uppsala University, BMC Box 582, SE-75123 Uppsala, Sweden; CNR, ITALY

## Abstract

The folding and unfolding of protein domains is an apparently cooperative process, but transient intermediates have been detected in some cases. Such (un)folding intermediates are challenging to investigate structurally as they are typically not long-lived and their role in the (un)folding reaction has often been questioned. One of the most well studied (un)folding pathways is that of *Drosophila melanogaster* Engrailed homeodomain (EnHD): this 61-residue protein forms a three helix bundle in the native state and folds via a helical intermediate. Here we used molecular dynamics simulations to derive sample conformations of EnHD in the native, intermediate, and unfolded states and selected the relevant structural clusters by comparing to small/wide angle X-ray scattering data at four different temperatures. The results are corroborated using residual dipolar couplings determined by NMR spectroscopy. Our results agree well with the previously proposed (un)folding pathway. However, they also suggest that the fully unfolded state is present at a low fraction throughout the investigated temperature interval, and that the (un)folding intermediate is highly populated at the thermal midpoint in line with the view that this intermediate can be regarded to be the denatured state under physiological conditions. Further, the combination of ensemble structural techniques with MD allows for determination of structures and populations of multiple interconverting structures in solution.

## Introduction

The folding of proteins to their functional conformations has been studied extensively both experimentally and through theoretical simulations. There has been great progress in understanding (un)folding reactions, in particular for small fast folding (*μ*s-ms) protein domains. Despite the apparent complexity, protein (un)folding reactions are usually fast (*μ*s-s) and often occur without accumulation of intermediates, which can be illustrated using a smooth funneled energy landscape [1–3]. Nevertheless, there are several cases where intermediates accumulate in protein (un)folding reactions as on-pathway species [4–8]. There is however an ongoing debate about whether these intermediates are productive in the strict definition that they are obligatory species on the path to the native state.

Among fast-folding proteins, *Drosophila melanogaster* Engrailed homeodomain (EnHD) is one of the best studied systems. A combination of experimental and computational methods have demonstrated that EnHD folds by initial formation of secondary structure elements that subsequently dock to form the native state [9, 10] in line with the diffusion-collision model [11]. Protein engineering in combination with nuclear magnetic resonance (NMR) showed that this (un)folding intermediate (or denatured state under physiological conditions, D_*phys*_) contains both native and non-native helices [12], and that the helix-turn-helix motif constituting H2-H3 forms independently of H1 [13], and can thus be regarded the main structural unit of the intermediate.

Small- to wide-angle X-ray scattering (S/WAXS) in solution has emerged as a powerful structural probe of biomolecules that explicitly side-step the fundamental limitations of conventional X-ray diffraction methods that probe proteins in the crystalline phase. It is in general not feasible to directly extract 3D atomic structures from disordered systems. However, by fitting S/WAXS data against structures obtained from X-ray crystallography, NMR spectroscopy and/or from theoretical modeling it is possible to extract information about the structural ensemble in the sample [14]. WAXS extends the data present in the conventional SAXS regime (*q* ∼ 0.3 Å^−1^), where information about shape and size of macromolecules can be obtained, out to a regime where scattering fingerprints from internal protein structures are present and can thus be viewed as a high-resolution extension of SAXS which provides low-resolution structural information of proteins [15]. Structural intermediates have uniquely been identified for smaller molecules in several time-resolved WAXS experiments [16–18] demonstrating the achievable resolution of the technique. WAXS has recently been extended to also probe the rearrangement of secondary structural elements within proteins [19]. In addition, a methodology based on refining the molecule of interest toward solution scattering data using MD simulation has very recently been developed and successfully applied on several molecular systems [20].

H^*N*^-N residual dipolar couplings (RDCs) are highly sensitive to the orientations of amide bond vectors within the molecular frame [21, 22]. Small changes in the relative bond vector orientation within that frame will result in a coupling different from that predicted from the reference structure. A poor correlation between measured and predicted data suggests a change in secondary or tertiary structures. The agreement of the experimental data set to the structure is evaluated using either a simple correlation coefficient (R) or, more commonly, using the Cornilescu Q factor (Q = rms(D_*calc*_-D_*obs*_)/rms(D_*obs*_), where rms, D_*calc*_, and D_*obs*_ represent root-mean-square deviation, predicted and observed RDCs, respectively) [23]. Solution structures corresponding to high resolution X-ray structures typically have Q < 0.25 [24].

In the present study, we investigate the evolution of the structural ensemble upon thermal denaturation of EnHD by an ensemble optimization algorithm that selects structures obtained from molecular dynamics (MD) simulations [25] and NMR [12] that together reproduce the S/WAXS data [25–27] at various temperatures: a rather similar approach was early proposed by Kozak *et al.* [28] We demonstrate the presence of three distinct ensembles of species during (un)folding of EnHD. These ensembles correspond to the denatured state D, an (un)folding intermediate I, corresponding to the denatured state under physiological conditions and the native state N. In particular, data at temperatures ranging from 20°C to 55°C suggest that the intermediate state becomes populated near the midpoint (apparent midpoint for thermal unfolding is about 52°C), rather than a more extended denatured state. We also find that the ensembles of substates within each population fit the S/WAXS data significantly better than for example the average NMR structure, thus capturing the flexible multi-state nature of proteins. The conclusions from S/WAXS and MD simulations are corroborated using residual dipolar couplings (RDCs) obtained from NMR experiments at different temperatures, showing the potential of the approach to detect and characterize protein (un)folding intermediates by X-ray scattering and MD simulations.

## Materials and Methods

### X-ray solution scattering

The S/WAXS experiments were carried out at the cSAXS beamline of the Swiss Light Source using a rapid-readout pixel detector, Pilatus [29]. The EnHD protein was expressed and purified as previously described [30]. A solution of 1.1 mM EnHD in 50 mM HEPES, 100 mM NaCl at pH 8.0 was delivered into the monochromatic X-ray beam (12.44 keV, ∼ 10^12^ photons/s in a 300 × 300 *μ*m spot) by being pumped through a 1 mm diameter (0.98 mm internal diameter) quartz capillary. Part of the tubing and the capillary was placed in-between two metal plates with a small hole for the X-ray to enter the capillary. The temperature was regulated by a thermo coupler and a cooling system integrated in the metal plates. About 30 cm of tubing on each side of the capillary was inserted in between the plates to ensure that the whole sample volume was heated to the desired temperature.

Pump triggering was integrated within the beam line control system, and for each acquisition cycle the sample was pumped continuously (2 *μ*l/s) to ensure that a new sample volume was exposed to X-ray for each measurement. During the acquisition cycle a sequence of scattering images were recorded using an integration time of 7 ms and a readout delay of 3 ms, equating to a readout frequency of 100 Hz. Pixel masking and radial integration of each Pilatus frame was computed at the beamline by in house software. A MATLAB script specifically written for this experiment was later utilized to filter out outliers (mainly caused by bubbles or aggregates in the solution), to normalize the intensity of the single azimuthally integrated frame to the incoming flux, for the statistical analysis, and to subtract the buffer signal from the protein solution.

In the scattering data there was some indications of aggregation (enhanced scattering at very low *q*), especially at elevated temperatures, and therefore a lower limit of *q* = 0.07 Å^−1^ was used in the structural analysis (*q* = 4*π* *sin*(*θ*)/*λ*, where 2*θ* is the scattering angle of incident X-ray beam and *λ* is the wavelength of the X-ray photons). Traditional Guinier plots, at *q* spanning from 0.01 to 0.1 Å^−1^ for proteins of this size, give the radius of gyration, i.e. the average dimension of the particles but not much information otherwise. At *q*-values below 0.07 Å^−1^ particle scattering will thus completely dominate and this region will be of less importance in the structural analysis performed in this work.

### Residual dipolar couplings

The H^*N*^-N residual dipolar couplings were measured as described previously [12] in 20 mM sodium acetate, pH 5.7. Briefly, the measurement was performed in radially compressed 7% acrylamide gels (37:1 bisacrylamide:acrylamide ratio) using the DSSE pulse program [31]. The data was analyzed using PALES software [32] using the 1ENH crystal structure as the template [33]. The conditions for the measurements are slightly different from those in the SAXS measurements in order to lower the stability of EnHD and ensure that the RDCs can be measured past the Tm of the protein on our Bruker DRX500 spectrometer equipped with a single axis gradient cryo-probe. Change in buffer condition causes decrease in Tm by about 10°C, yet since there are no histidine residues and all basic or acidic side chains are well solvated, there is no reason to assume the protonation state of the protein would change significantly.

### Molecular dynamics simulations

MD simulations of the EnHD (PDB ID: 2JWT) were carried out based on the first NMR model of EnHD in the PDB file, i.e. the most optimized structure. Simulations were performed using the GROMACS package [34] and the AMBER99SB-ILDN force field [35] with the TIP3P water model [36]. The protein molecule was immersed in a periodic box containing 32759 water molecules. Additionally, 60 Na+ and 68 Cl- were added to reach a salt concentration of 50 mM corresponding to the experimental value and neutralizing the positive protein charge. The box size was ∼ 10 nm which prevented protein interactions through periodical boundaries even in the unfolded state. A cutoff distance 1 nm has been used for calculations of Lennard-Jones interactions and PME was used for the treatment of the long-range electrostatic interactions. An integration time step of 2 fs was used and the Berendsen algorithm for temperature and pressure control [37] was used with 0.1 and 1 ps coupling constants, respectively. The pressure was kept at 1 atm. In total eight trajectories of each 100 ns were generated. The temperature for each production run was 275, 300, 325, 350, 375, 400, 450 and 500 K. Before the production run, the system was minimized using steepest descent for 1000 steps after which the system was equilibrated in dynamic simulation 20 ps long with 1 fs time step at 275 K. Atomic coordinates from the last equilibration snapshot were then used as an input for the production runs at different temperature.

### Cluster analysis

Protein structures for fitting to the experimental data were obtained after cluster analysis on each MD trajectory. Clustering has been accomplished by the algorithm due to Daura et al. [38] which is implemented in the GROMACS package [34]. Structures for the cluster analysis were sampled from MD-trajectory every 10 ps, for a total of 10000 structures from each trajectory. As the clustering criterion, the root mean square deviation (RMSD) of main-chain and C-beta atoms was used. Cut-off clustering distances of 1.0, 2.5 and 3.5 Å have been used. Only clusters consisting of ten or more structures have been taken into account for SAXS fitting. The representative structure for each cluster was taken to be the structure from the MD-trajectory most closely located to the center of the cluster in RMSD space.

### Computations of X-ray scattering spectra and NMR restraints

Theoretical spectra of X-ray scattering and violations from NMR restraints were calculated from the central cluster structures. X-ray scattering was computed using the CRYSOL [39] software in the range of reciprocal space corresponding to the experimental data. The solvent was generated implicitly for such computations with the density set to 340 e/nm^3^ corresponding to the experimental value. Due to the small size of the protein and fast sidechain dynamics, the solvent shell contrast around the protein has been kept to zero [14]. The maximum order of harmonics, which defines the resolution of the scattering curve, was set to seven. The other configuration parameters of CRYSOL were set to the default values.

Atomic distances have been calculated directly from the protein structure by g_disre program [40], which is a part of the GROMACS package. EnHD is in fast exchange in NMR chemical shift timescale, since it is an ultrafast folder (> 100 000/s), those RDCs and NOEs could be interpreted as being averaged over all the populated conformers. Ensemble-averaged NMR violations have been calculated based on experimental distance restraints [12] and atomic distances averaged over all conformations *i* of the ensemble by applying power-averaging, *d* = (∑(*d*_*i*_)^−*n*^)^−1/*n*^. The power *n* is usually taken between three and six, where the higher number gives better correlation between calculated and experimental distance restrains [41]. In the current work *n* = 3 was selected.

### Optimization algorithm

The ensemble optimization approach by Bernado and co-workers [42] was utilized to obtain the ensemble of protein structures that reproduced the experimental scattering data using an iterative genetic algorithm [43]. Ensembles were formed from an extensive pool of conformers generated by MD simulations. The fitting was performed to reproduce the logarithm of the experimental scattering intensity, log I(q). The square difference between experimental and ensemble-averaged X-ray scattering was used as the target function in the fitting. At each step of the algorithm several ensembles of structures, denominated chromosomes, were tested and sorted according to the value of the target function. The specified number of ensembles showing the lowest values of target function was selected to pass into the next iteration. The size of each ensemble had a maximum of 20 spectra. All spectra were of equal weight in the chromosome, yet multiple repetitions of structures were allowed, which gives the possibility to change the partial weights of the structures that contributes to the ensemble. Optimization was performed for 10000 iterations and repeated 50 times. In total 20 ensembles were selected to pass through each iteration of the algorithm. The crossover operator was tuned to generate the same number of chromosomes as the initial population. The number of chromosomes generated by the mutation operator exceeded the number of initial chromosomes by the factor of two. This last option was selected in order to achieve rapid convergence toward the optimal solution due to higher ensemble divergence created by the mutation operator. The code for the optimization algorithm was implemented in a MATLAB package and is available from the authors upon request.

## Results

### Molecular dynamics simulations for sampling

**Fig 1:**
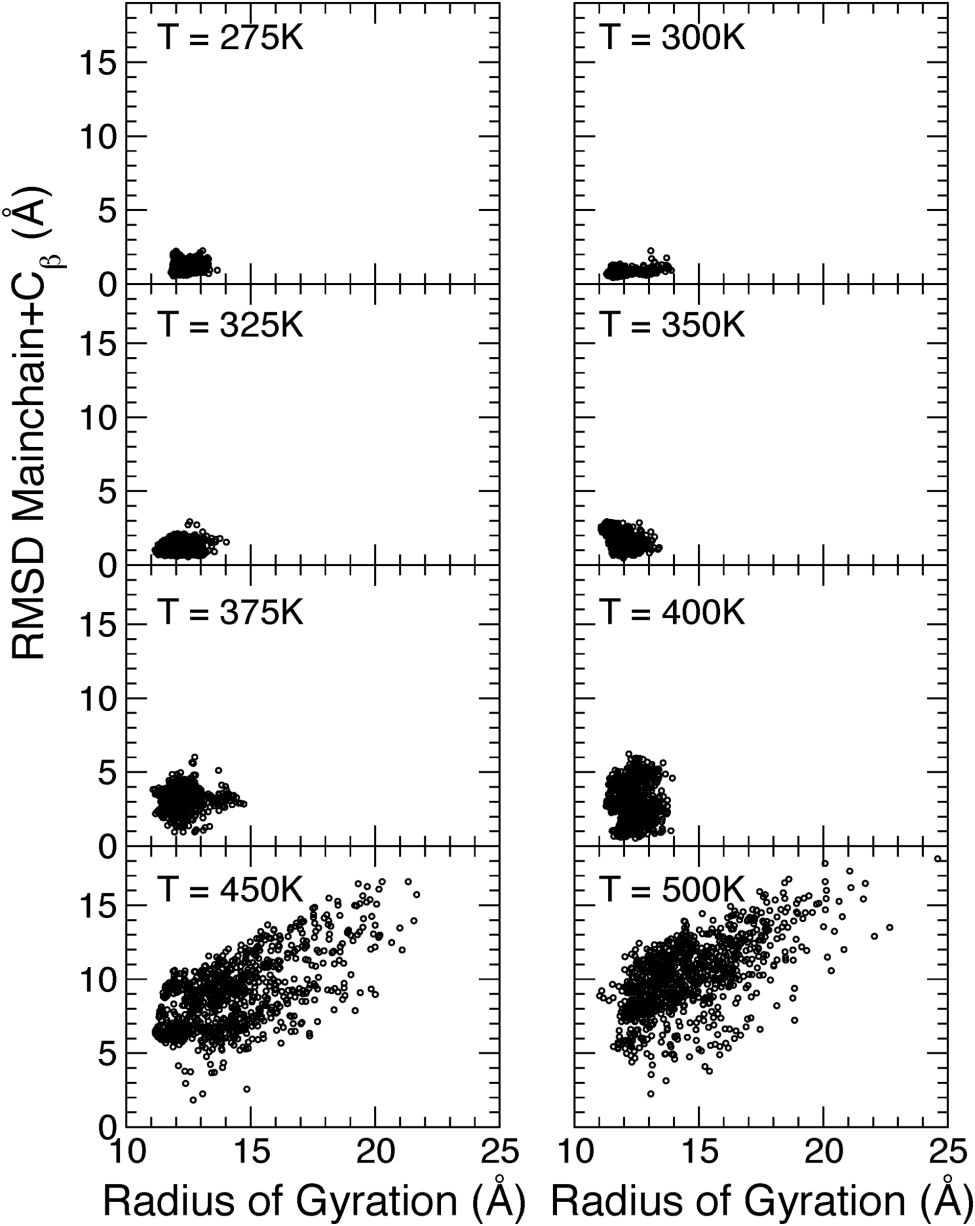
Conformational sampling of EnHD at 8 different temperatures based on MD simulations. The plot also shows the correlation between radius of gyration and root mean square deviation from the NMR structure. The RMSD have been calculated for the mainchain and C_*β*_ atoms of all 61 residues.

MD simulations are a powerful tool to generate physical conformations of biomolecules. The part of phase space that is sampled depends predominantly on the temperature applied. The conformations sampled in simulations of EnHD at 8 different temperatures were projected on a plane with the radius of gyration on the X-axis and the root mean square deviation from the NMR structure on the Y-axis (Fig 1). An interesting observation is that there are structures that deviate up to 10 Å from the experimental structure of the native state and that are nevertheless compact; that is, non-native structures of EnHD do not have to be extended. Thus, the simulations show a large diversity of compact structures in the molecular species resulting from thermal (un)folding. The highest temperatures yield the most unfolded structures within the relatively short (100 ns) simulation time. Since the structures are generated just for sampling phase space, the fact that the temperatures of the simulation are much higher than those used in the experiments is irrelevant.

### Modeling the structural ensembles from S/WAXS data

Conventional SAXS experiments give information mainly about the overall shape and size of proteins in solution. By extending the collected scattering angles, into the range between 0.4 and 0.7 Å^−1^ where scattering more specifically related to secondary and tertiary protein structures will be present, the conformational ensemble present in the sample can be characterized. In an optimization procedure [42], based on an iterative genetic algorithm [43], the ensemble of protein structures that best reproduced the experimental data was selected from a pool of MD and NMR structures as is further discussed in Materials and Methods.

**Fig 2:**
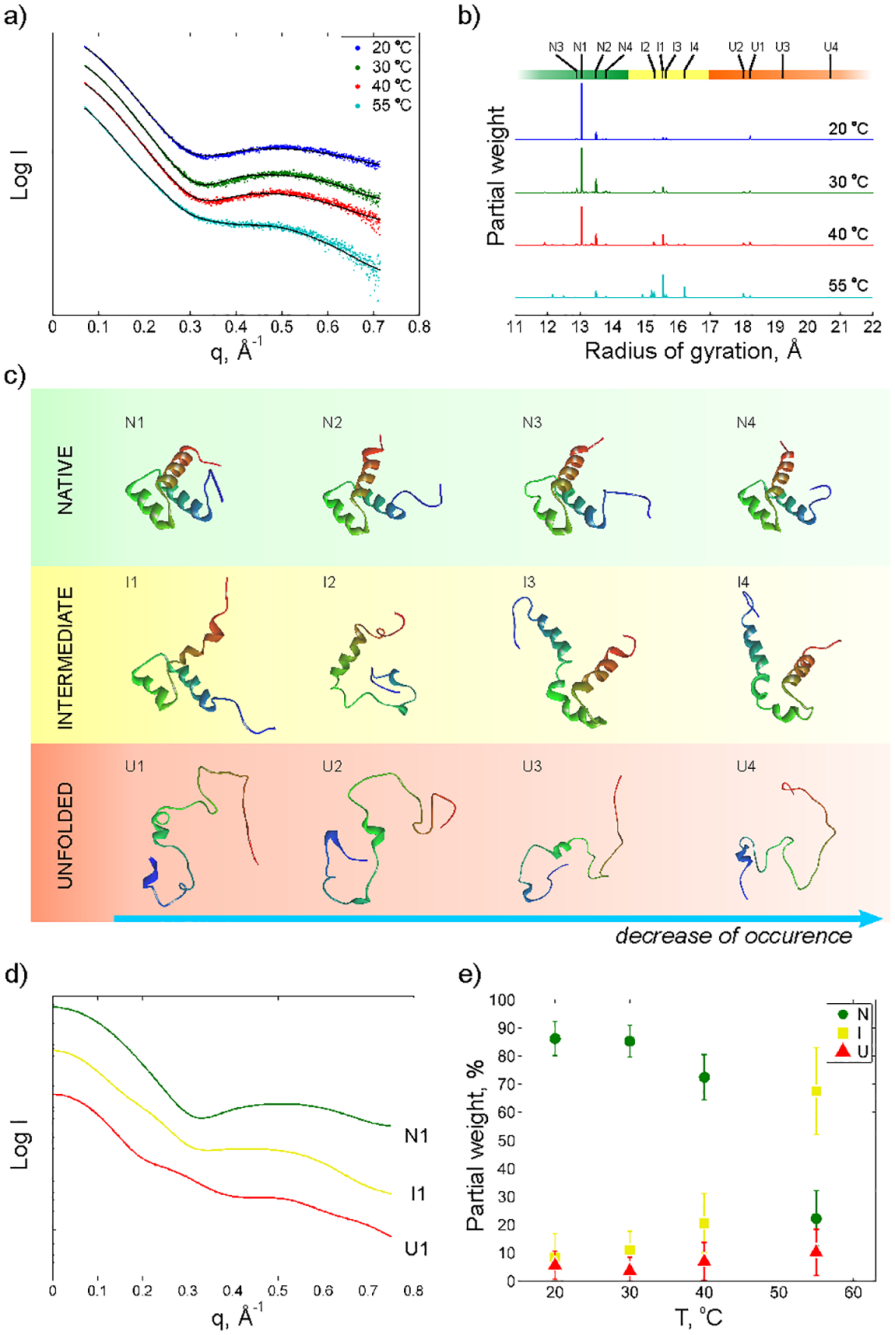
X-ray scattering intensity as a function of the scattering vector q at four different temperatures. b) histogram of radius of gyrations of proteins obtained from the optimization procedure at the different temperatures, c) the four most populated structures for native, intermediate and unfolded as determined by a cluster analysis, d) simulated scattering curves for the most populated native (N1), intermediate (I1) and unfolded conformations (U1) and e) population of native (N), intermediate (I) and unfolded (U) as a function of temperature. The error bar at e) indicates a standard deviation obtained in ensemble fitting. Scattering profiles at a) and d) and weight distribution at b) are shifted to increase visibility.

X-ray scattering (0.01 < *q* < 0.7 Å^−1^) was collected from a 1.1 mM sample of EnHD at four temperatures, 20, 30, 40 and 55°C (Fig 2a) in an attempt to structurally resolve conformational changes in the folding/unfolding process of the protein.

The scattering profiles (Fig 2a) show a pronounced broad peak at around 0.5 Å^−1^ that fades away with temperature. This pattern provides the signature of a significant shift in the protein population towards less folded structures at the melting point.

Fig 2b shows the radius of gyration of the protein structure ensemble obtained from the fitting to the scattering data using the optimization algorithm (see Materials and Methods). Three groups of structures can be identified. The first group located around 13 to 14 Å (green) contains native-like structures which dominate at low temperatures. The second group (yellow) contains proteins with less organized structures with radii of gyration between 14.5 and 17 Å. The third group (red) at higher radii of gyration contains denatured structures where some of the helices are completely unfolded. In Fig 2c a structural comparison of the four most abundant structures in each group is displayed. The numbering within each group is related to the relative weight of these structures in the fitting at most temperatures. The N1 structure, which is selected with high abundance in the native group, is the 12^*th*^ structure from the NMR ensemble consisting of 25 structures (PDB ID: 2JWT [12]). Fig 2d shows the calculated scattering profiles for the most abundant structures in each group. A clear difference in scattering signatures between the three groups of structures is apparent, and it is obvious that the structure seen around 0.5 Å − 1 in the experimental data at low temperature is related to a more folded structure while an unfolded structure gives a more straight scattering profile. In Fig 2e the relative weight of the three groups at the four temperatures is shown. As expected, a transition from native like structures towards more unfolded structures with increasing temperature is observed.

### Validation of the structural ensemble using RDCs

Prior comparison of the solution NMR parameters of EnHD to the X-ray data revealed an outstanding agreement (Cornilescu Q factor < 0.25) of the EnHD H^*N*^-N RDCs when the protein was folded [12]. For a fully unfolded protein, the H^*N*^-N RDCs in radially compressed acrylamide gels will be slightly negative, and there will be no agreement between the measured RDCs and the crystal structure across all of the protein [44].

**Fig 3:**
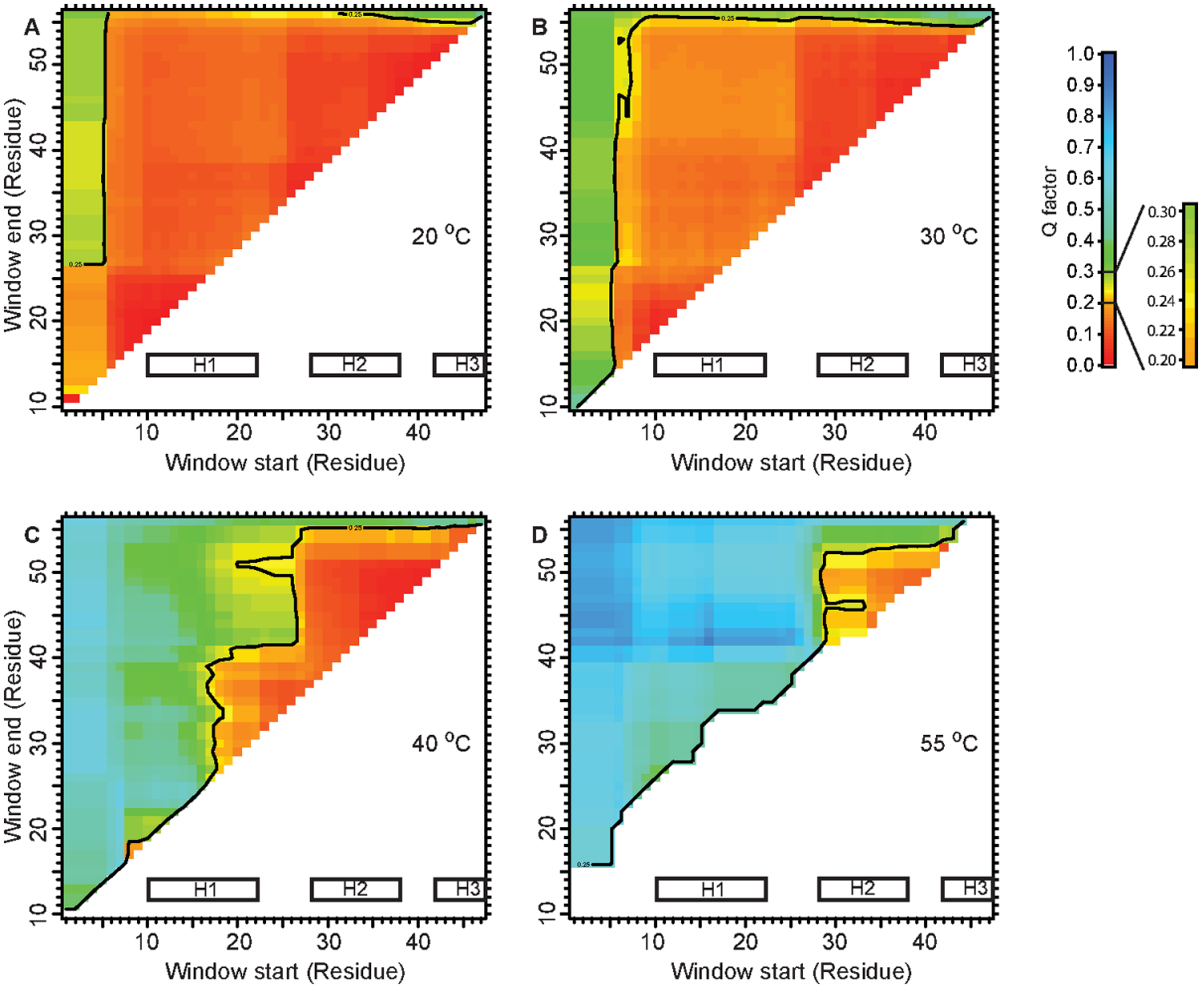
Agreement of H^*N*^-N RDCs to EnHD crystal structure at increasing temperatures. The Cornilescu Q factors were calculated using a sliding window from ‘Window start’ to ‘Window end’ to estimate the agreement of the given residue range. Q = 0.25 was used as the cut-off for the agreement [24]. At 20°C (a) and 30°C (b) EnHD is fully folded, and any chosen residue window displays perfect agreement with the crystal structure. At 40°C (c) the agreement for the 10–55 fragment is not present, but the HTH motif remains structured. This indicates that H1 must be undocked from H2/H3. C. At 55°C (d) around the thermal midpoint of denaturation, some H^*N*^-N RDCs for H2/H3 remain in agreement with the crystal structure.

As we measured the RDCs at increasing temperatures for EnHD, we observed that while the agreement for the 10–55 residue range, which encompasses H1/H2/H3 was lost, the values were still consistent with a preserved HTH motif at 40°C (Fig 3b). Even at 55°C, close to the apparent midpoint for thermal denaturation [9], the agreement of the H^*N*^-N RDCs to the crystal structure for the 28–52 residue fragment was as good, suggesting a significant population of the HTH motif (Fig 3d).

## Discussion

The ensemble optimization of conformations generated by MD simulations (Fig 1) against the S/WAXS data allows for deconvolution of structures and populations of multiple interconverting structures in solution. This gives new insights into the structural complexity of protein (un)folding in Engrailed Homeodomain. The obtained S/WAXS structures at low temperatures are in good agreement with those obtained in previous NMR studies [12] while the structures at temperatures close to the thermal midpoint (Fig 2) are compatible with the H^*N*^-N residual couplings (Fig 3).

From the fitting of the X-ray scattering data we can resolve three groups of structures that seem to be present at all temperatures but with varying occupancy. Few structures are presented in the optimized ensembles, which is not surprising due to high stability of the protein and fast convergence of the optimization algorithm. As expected, the occurrence of the native-like structures will strongly decrease at the thermal midpoint, from almost 90% to 20%. At 55°C an intermediate structure, I1, present already at low temperature, will increase in abundance while some more unfolded intermediate structures (I2 and I4) appear as well. It is particularly interesting to note that at 55°C the population of intermediate and denatured states contains a high degree of secondary structure.

At the lowest temperatures one of the NMR structures [12], N1, was selected with a partial weight of 65% in the fitting, demonstrating that the S/WAXS data is consistent with the known experimental structure of EnHD and, of significance for the whole analysis, that the optimization algorithm works reliably. The other three structures in the native group are quite similar to the NMR structure where the main differences are located at the termini. In the intermediate group the helices are usually maintained with a few exceptions such as I2 for which two of the helices, H1 and H2, are more or less unfolded. Less than 6% of the total ensemble at 20°C consists of unfolded structures.

The averaged RMSD for S/WAXS-fitted ensembles is in the range of 1.1 to 3.5 Å, as shown in Table 1), which is comparable with structural ensembles defined by 2D NOESY NMR [12]. The number of violated restraints for 20, 30 and 40°C are below 1%. Similar number of violated restraints was obtained for NMR-refined and crystal structures [12]. Larger structural ensembles applied for calculation of NMR restrains violations slightly improve the number of violated restrains [45], yet fitting of SAXS data with such ensembles is computationally expensive. In addition, the use of only the NMR structures in the optimization against the X-ray data results in a much poorer fitting with about 50% larger *χ*-value, which again indicates the presence of additional structures in the sample than those recovered from NMR structural ensemble [12].

**Table 1:** The number of violated restraints according to Fletcher et al. [45] The total number of NOE restraints is 735. The RMSD have been averaged over all structures in the S/WAXS refined ensemble.

Experimental temperature	Violated restraints	RMSD, Mainchain + C_*β*_, Å
20°C	3(< 0.5%)	1.1
30°C	2(< 0.5%)	1.1
40°C	3(< 0.5%)	1.8
55°C	68(9%)	3.5

It was previously shown that the H2-H3 helix-turn-helix motif of EnHD folds independently of H1 and with a rate constant similar to the fast phase for folding of full-length EnHD [13]. This, and data on the destabilized L16A mutant of EnHD, which is molten-globule like under physiological conditions due to the lost tertiary contact at the 16^*th*^ residue [46], make a strong case for the H2-H3 motif being present in the folding intermediate and/or the denatured states. The present data provide even more detail. Firstly, the proposed H2-H3 motif is present in some of the intermediate structures (I3 and I4, Fig 2e) but not in the other two. The RDC matrix at 40°C also suggests that the interaction between H1 and H2/H3 is weak. Note that the RDC plots present an ensemble average and hence the result at 40°C qualitatively agrees with structure analysis based on S/WAXS and MD. Secondly, the unfolded structures (Fig 2e) are relatively compact as well. Thirdly, the structures of the intermediates at 55°C contain a significant fraction of alpha helix, suggesting they could be characterized as native-like in circular dichroism experiments for instance, and give an apparent higher population of native-like structures in such an analysis.

Protein folding is a complex process and sometimes intermediate states that may be detected using one technique cannot be observed using other means. Here we have shown that by combining experimental techniques with theoretical modeling it is possible to extract new information from even a well-studied protein like EnHD. Simplified descriptions of protein folding in terms of funnels [1, 2] have been useful in formulating some general principles of folding. However, atomic-level descriptions of protein folding pathways are dominated by specific interactions within proteins and between protein and solvent and require theoretical simulations that use a realistic description of protein and solvent [47–53]. On the experimental side, local interactions require elucidation at the atomic and near atomic levels by such techniques as NMR [54, 55] and by Φ-value analysis [56]. Where possible, multiple complementary experimental techniques should be used that give further local and global information on structure. From the native state of a protein the folding road is uphill. Progress in studying protein folding under physiological conditions and in the context of, for instance, misfolding-induced disease depends on those methods and ideas [57]. Further, the methodology used here should not only be restricted to folding but should, in principle, be applicable in any determination of protein structures and populations of multiple interconverting structures in solution.
